# Structural analysis of causal pathways between adherence, satisfaction and clinical outcomes in hypertensive patients using a mobile adherence intervention: A SEM–NCA–cIPMA approach

**DOI:** 10.1038/s41371-026-01132-x

**Published:** 2026-03-27

**Authors:** Rajat Rana, Baharudin Bin Ibrahim, Hasniza Binti Zaman Huri, Izyan Binti A. Wahab, Kayatri Govindaraju, Mohd. Syamir Mohamad Shukeri, Siew Chin Ong

**Affiliations:** 1 Department of Clinical Pharmacy and Pharmacy Practice, Faculty of Pharmacy, Universiti Malaya, 50603 Kuala Lumpur, Malaysia; 2 Department of Pharmaceutical Life Sciences, Faculty of Pharmacy, Universiti Malaya, 50603 Kuala Lumpur, Malaysia; 3 School of Pharmaceutical Sciences, Universiti Sains Malaysia, 11800 Penang, Malaysia

**Keywords:** Preventive medicine, Hypertension

## Abstract

Poor medication adherence remains a major barrier to effective hypertension control, particularly in low-resource settings. This study aimed to explore causal pathways, necessity conditions and optimization priorities of the CareAide mobile application for improving medication adherence and clinical outcomes among hypertensive patients. A prospective, randomised, open-label, two-arm trial was conducted at University Malaya Medical Centre. Adults (N = 275) with hypertension and low Morisky Medication Adherence Scale (MMAS-8) scores were randomised to CareAide plus usual care or usual care alone for six months. Primary outcome was adherence (MMAS-8) at third (F1) and sixth month (F2); secondary outcomes included systolic/diastolic blood pressure (Hypertension F1, F2) and application satisfaction via Mobile Adherence Satisfaction Scale (MASS). Structural Equation Modelling (SEM), Necessary Condition Analysis (NCA) and Combined Importance–Performance Map Analysis (cIPMA) tested hypotheses from an integrated theoretical framework. Intervention showed large positive effects on adherence at (F1 β = 0.58, F2 β = 0.79, *P* < *0.001*). Six-month adherence mediated the intervention’s impact on blood pressure (indirect β = −0.16, *P* = *0.006*) and together with early adherence, formed a sequential mediation chain (β = −0.05, *P* = *0.01*). NCA showed the intervention (d = 0.18) and early adherence (d = 0.28) were necessary for high F2 adherence; the intervention (CE-FDH = 100%) and prior blood pressure control (CE-FDH = 48.58%) were necessary for optimal F2 blood pressure. Feature satisfaction (FS) was both sufficient (β = 0.20, *P* < *0.001*) and necessary (d = 0.29) for sustained adherence. cIPMA identified the intervention, early adherence and FS as high-importance, low-performance targets. CareAide significantly improves adherence and blood pressure via a temporally sequenced behavioral pathway with early adherence gains and FS are critical, actionable factors. **Trial registration:** CAREAide Trial (ClinicalTrials.gov identifier: NCT06068309).

## Introduction

Medication non-adherence remains a critical barrier to optimal chronic disease management, particularly among patients with hypertension, where poor adherence contributes to inadequate blood pressure control, increased hospitalization rates and elevated healthcare costs. In Malaysia, adherence rates among hypertensive patients remain suboptimal, despite pharmacological advancements and widespread access to public healthcare facilities [1]. While conventional strategies such as physician counselling and pill organizers have shown limited and inconsistent impact, recent years have seen growing interest in mobile health (mHealth) interventions, which promise scalable, patient-centered solutions to improve adherence.

Despite the proliferation of mHealth applications globally, few interventions have undergone rigorous, theory-driven evaluation using multidimensional outcome frameworks in middle-income contexts [2]. Moreover, limited empirical clarity exists on the mechanisms through which these digital interventions influence adherence behaviors and downstream clinical outcomes [3].

To address these gaps, the CAREAide trial adopted a multimethod analytic framework that integrated Structural Equation Modeling (SEM), Necessary Condition Analysis (NCA) and Combined Importance–Performance Map Analysis (cIPMA) to evaluate the sufficiency of the hypothesized causal pathways, identify indispensable threshold conditions for adherence and clinical improvement and prioritize determinants for strategic optimization. This study aimed to explore the causal pathways underlying a digital adherence intervention’s effectiveness including temporal mediation by early adherence identify necessary conditions for optimal outcomes and prioritize optimization targets via user satisfaction and sociodemographic moderators.

## Theoretical background

**Fig. 1:**
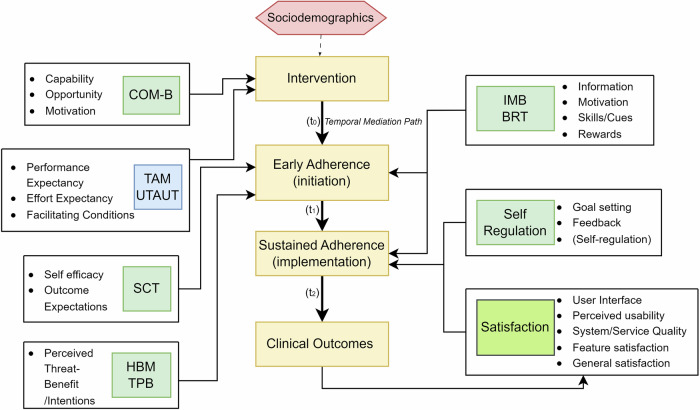
Integrated Theoretical Framework for Mobile Adherence Interventions. A conceptual diagram synthesizing COM-B, SCT, IMB, TAM/UTAUT and socio-ecological moderators used for developing Mobile Adherence Interventions. Arrows indicate hypothesised causal paths.

This study is grounded in an integrative theoretical framework that synthesizes models from health behavior change, technology acceptance and socioecological systems to explain how the CareAide mobile health intervention is hypothesized to improve medication adherence and clinical outcomes in patients with chronic diseases (Fig. 1). Recognizing the complexity of adherence behavior, the framework incorporates both psychological and technological determinants, while also accounting for the dynamic and moderated nature of intervention effects over time.

At the behavioral level, the COM-B model serves as a foundational framework, positing that Capability, Opportunity and Motivation are necessary for behavior change [4]. The CareAide application is designed to enhance psychological capability (e.g., via intuitive medication scheduling), physical opportunity (e.g., reminders and cues), and both reflective and automatic motivation (e.g., through progress feedback and habit reinforcement). These elements work synergistically to reduce common adherence barriers such as forgetfulness and regimen complexity. Social Cognitive Theory (SCT) suggests that health behaviors are shaped through dynamic interactions between personal cognitions (self-efficacy, outcome expectations), environmental cues, and reinforcement mechanisms [5]. Within this model, mobile health applications like CareAide function as environmental facilitators that strengthen self-efficacy via medication reminders (cues to action), adherence tracking (self-monitoring) and feedback (reinforcement), thereby promoting behavioral initiation and maintenance. This suggests the direct intervention effects on adherence and clinical outcomes. This behavioral model is complemented by the Health Belief Model (HBM) and Theory of Planned Behavior (TPB), which emphasize the role of perceived threat, benefits and self-efficacy in guiding health actions [6, 7]. The application’s features, such as real-time tracking and reminders, act as external cues that enhance perceived control and intention to adhere. In addition, Self-Regulation Theory supports the intervention’s feedback mechanisms, which enable users to set adherence goals, monitor their progress and adjust behaviors accordingly, facilitating long-term maintenance of adherence [8].

The Information–Motivation–Behavioral Skills (IMB) model further explains how the application provides structured information, enhances motivational salience through satisfaction and reinforcement, and cultivates behavioral skills necessary to implement consistent medication-taking behavior [9]. Alongside this, Behavioral Reinforcement Theory (BRT) proposes that cues and rewards embedded in the application promote operant conditioning, reinforcing adherence habits over time [10]. To achieve its intended effect, the intervention must also be accepted and used consistently, a process explained by the Technology Acceptance Model (TAM) and Unified Theory of Acceptance and Use of Technology (UTAUT) [11, 12]. UTAUT identifies performance expectancy (perceived utility), effort expectancy (user interface intuitiveness) and facilitating conditions (system/ service quality) as critical determinants of technology adoption. Higher user satisfaction is theorized to mediate the effect of the intervention on sustainable adherence and clinical outcomes [13].

A temporal mediation paradigm frames adherence as a phased process wherein initiation (3-month adherence) precedes implementation (6-month adherence). This conceptualizes short-term adherence gains as foundational to long-term habits, justifying the causal pathway where early adherence mediates the intervention’s effect on later adherence and subsequent clinical outcomes. This sequential mechanism aligns with EMERGE guidelines, which distinguish between initiation and implementation phases of adherence [14]. This aligns with contemporary perspectives on adherence as a dynamic, evolving behavior [15]. Finally, the framework incorporates Socioecological Theory, which acknowledges that the effectiveness of digital interventions is moderated by demographic and contextual factors such as age, income, education and digital access [16]. For instance, higher education or income may enhance app utilization through digital literacy, while gender, weight or age may introduce differential barriers to engagement [17]. These variables influence intervention reach and efficacy, serving as boundary conditions for equitable implementation.

## Conceptual framework and hypotheses development

**Fig. 2:**
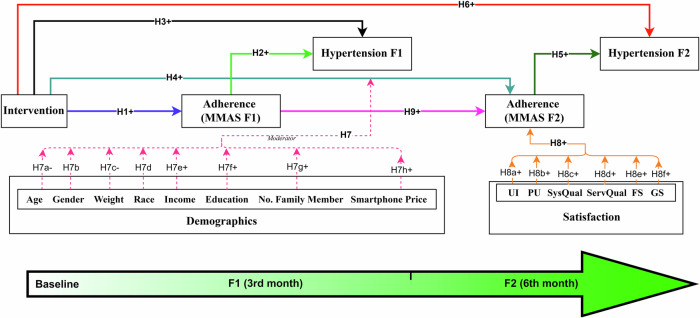
Proposed Conceptual Model. Structural model used in PLS-SEM showing the intervention → MMAS F1 → MMAS F2 → Hypertension F2 pathway, satisfaction domains as predictors of MMAS F2 and demographic moderators on the Intervention→MMAS F2 path.

Based on the integrative theoretical framework described above, the intervention is hypothesized to improve short-term adherence at 3 months (MMAS F1; H1 + ), which in turn enhances immediate clinical outcomes (Hypertension F1; H2 + ) and predicts long-term adherence at 6 months (MMAS F2; H9 + ). This progression is expected to mediate long-term clinical outcomes (Hypertension F2) as tested in H10+ to H13 + . Direct effects of the intervention on clinical outcomes at both time points (H3 + , H6 + ) acknowledge additional behavioral mechanisms. User satisfaction domains like usability, performance, system/service quality and motivational salience are postulated to sustain adherence (H8 + ), while sociodemographic moderators (H7a–H7h) capture contextual variability. Overall, the hypotheses test a temporally sequenced, theory-driven model of digital health behavior change (Fig. 2) (Table 1).

**Table 1:** Proposed Hypotheses.

	Hypothesis Statement
H1+	Intervention has a positive effect on medication adherence at 3^rd^ month (MMAS F1).
H2+	Medication adherence at 3^rd^ month (MMAS F1) is positively associated with improved clinical outcomes at 3^rd^ month (Hypertension F1).
H3+	Intervention has a direct positive effect on clinical outcomes at 3^rd^ month (Hypertension F1).
H4+	Intervention has a positive effect on medication adherence at 6^th^ month (MMAS F2).
H5+	Medication adherence at 6^th^ month (MMAS F2) is positively associated with improved clinical outcomes at 6^th^ month (Hypertension F2).
H6+	Intervention has a direct positive effect on clinical outcomes at 6^th^ month (Hypertension F2).
H7	Demographics (Age: H7a-, Gender: H7b, Weight: H7c-, Race: H7d, Income: H7e + , Education: H7f + , Number of family members: H7g + , Smartphone price: H7h + ) positively moderate the relationship between intervention and medication adherence at 6^th^ month (MMAS F2).
H8+	Satisfaction (UI: H8a + , PU: H8b + , SysQual: H8c + , ServQual: H8d + , FS: H8e + , GS: H8f + ) has a positive effect on medication adherence at 6^th^ month (MMAS F2).
H9+	Medication adherence at 3^rd^ month (MMAS F1) has positive effect on medication adherence at 6^th^ month (MMAS F2).
H10+	The effect of the intervention at 6^th^ month medication adherence (MMAS F2) is mediated by 3^rd^ month medication adherence (MMAS F1).
H11+	The effect of 3^rd^ month medication adherence (MMAS F1) on 6^th^ month clinical outcomes (Hypertension F2) is mediated by 6^th^ month medication adherence (MMAS F2).
H12+	The effect of the intervention at 6^th^ month clinical outcomes (Hypertension F2) is mediated by 6^th^ month medication adherence (MMAS F2).
H13+	The effect of the intervention on clinical outcomes at the 6^th^ month (Hypertension F2) is positively mediated through medication adherence at the 3rd month (MMAS F1) and at the 6^th^ month (MMAS F2).

## Methods

### Study design

The CAREAide trial was designed as a prospective, randomized, open-label, unblinded, two-arm, parallel-group study with an intent-to-treat framework, among patients with chronic conditions in the outpatient clinics of University Malaya Medical Centre (UMMC), a tertiary healthcare institution located in an urban area of Malaysia. This study was registered as CAREAide Trial in ClinicalTrials.gov (NCT06068309). The study adhered to the EMERGE guidelines proposed by ESPACOMP, thereby evaluating adherence at both the initiation and implementation phases [18]. Participants were monitored at three time points: baseline (B) and a follow-up at 3^rd^ month (F1) and at 6^th^ (F2) post-enrolment. Ethical approval for the study was granted by the institutional medical research ethics committee.

### Ethics approval and consent to participate

All methods performed in this study involving human participants were conducted in accordance with the ethical standards of the University Malaya Medical Centre Medical Research Ethics Committee and with the 2013 revision of the Declaration of Helsinki [19]. Ethical approval was obtained from the University Malaya Medical Centre Medical Research Ethics Committee, Universiti Malaya, Kuala Lumpur, Malaysia (MREC ID: 022311-11069). Written informed consent was obtained from all participants prior to enrolment in the study. The trial was registered at ClinicalTrials.gov (NCT06068309).

### Participants

This study was conducted in accordance with the CONSORT guidelines [20]. Eligible participants were adults aged 18 years or older who had been clinically diagnosed with hypertension for a minimum duration of six months. Inclusion criteria required that participants exhibit low medication adherence, defined by a Morisky Medication Adherence Scale (MMAS) score below 6 and be prescribed at least three medications daily or two medications administered at multiple dosing intervals. Exclusions included terminal illness, cognitive impairment or use of another adherence application. All participants were recruited from outpatient clinics at University Malaya Medical Centre (UMMC) and ownership of a smartphone with an active internet connection was mandatory for participation. Individuals who were currently using other adherence-related mobile applications or had terminal illnesses were excluded from the study. After receiving participants consent, they were randomly assigned in a 1:1 ratio to either the intervention group, which received the CareAide mobile application in addition to routine care or the control group, which continued with standard care alone.

### Intervention

**Fig. 3:**
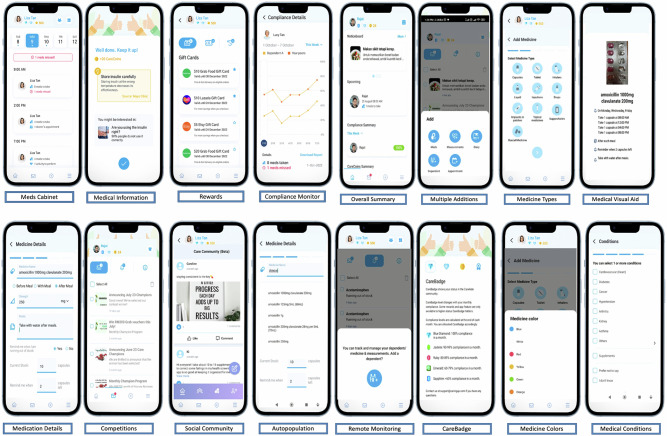
CareAide Application Features. Interface of CareAide mobile appliation featuring Home dashboard (medication schedule, adherence summary), Reminder setup screen (Meds Cabinet, Medicine addition), Behavioural Modifying featues (Medication feedback, Rewards, Social Community, Remote Monitoring).

Participants assigned to the intervention group (IG) were provided with support and instructions to install and operate the CareAide mobile application on their smartphones. They were instructed to utilize the application for medication management over a duration of six months. In contrast, individuals in the control group (CG) continued receiving routine clinical care as directed by their healthcare providers. A follow-up evaluation was conducted at the end of the sixth month post-enrolment for both groups to assess medication adherence and relevant clinical parameters. The CareAide application was equipped with several key functionalities, including automatic input of prescribed medications, customizable reminder systems, auditory notifications and adherence monitoring tools (Fig. 3).

### Outcome measurements

The principal outcome of this study was medication adherence, evaluated using the 8-item Morisky Medication Adherence Scale (MMAS-8), a psychometrically validated instrument recognized for its strong internal consistency (Cronbach’s α = 0.83), high sensitivity (93%) and moderate specificity (57%) [21, 22]. The scale yields scores ranging from 0 to 8, with higher scores reflecting greater adherence. Assessments were conducted at baseline and at the 3^rd^ month (F1) and 6^th^ month (F2) follow-up. Secondary outcomes comprised clinical indicators relevant to hypertension management, specifically systolic and diastolic blood pressure (SBP and DBP) [23]. In addition to adherence and clinical measures, participant satisfaction with the mobile intervention was assessed using the Mobile Adherence Satisfaction Scale (MASS), a psychometrically validated tool which comprised 26 items across six dimensions (user interface (UI), perceived usability (PU), system quality (SysQual), service quality (ServQual), feature satisfaction (FS) and general satisfaction (GS)), demonstrating excellent reliability (Cronbach’s α = 0.89) and strong model fit (KMO = 0.84; CFA: CR > 0.70, AVE > 0.50). The scale yields scores ranging from 1 to 5, with higher scores reflecting greater satisfaction [24].

### Statistical analysis

A priori power analysis indicated that 240 participants (120 per group) were required to detect a medium effect size (Cohen’s *d* = 0.40) with 80% power at two tailed α = 0.05, accounting for 20% attrition [25]. Additionally, the final sample size was consistent with widely accepted guidelines for factor analysis, which recommend a respondent-to-item ratio of approximately 5 to 10 participants per item [26]. Given that our scale comprised of 22 items, the minimum required sample size ranged from 110 to 220.

Analyses followed the intention-to-treat principle. Descriptive and inferential statistics were conducted using SPSS v29, while Structural Equation Modeling (PLS-SEM), Necessary Condition Analysis (NCA) and Combined Importance–Performance Map Analysis (cIPMA) were performed in SmartPLS 4. SEM assessed direct, indirect and mediated effects via 5000-sample bootstrapping, with model fit and validity evaluated using standard PLS-SEM criteria [27]. We estimated direct, indirect and serial mediation paths from intervention → MMAS F1 → MMAS F2 → Hypertensive outcomes. NCA identified essential conditions for adherence and clinical outcomes by assessing bottleneck thresholds, while IPMA prioritized factors leading to improved adherence and clinical outcomes based on their importance and performance with performance indices (0–100) and NCA necessity (CE-FDH, d) to prioritize levers for MMAS F2 and BP at F2. Sociodemographic moderation was tested and missing data were handled via multiple imputation. Significance was set at *P* < *0.05* (two-tailed).

## Results

### Baseline characteristics

**Fig. 4:**
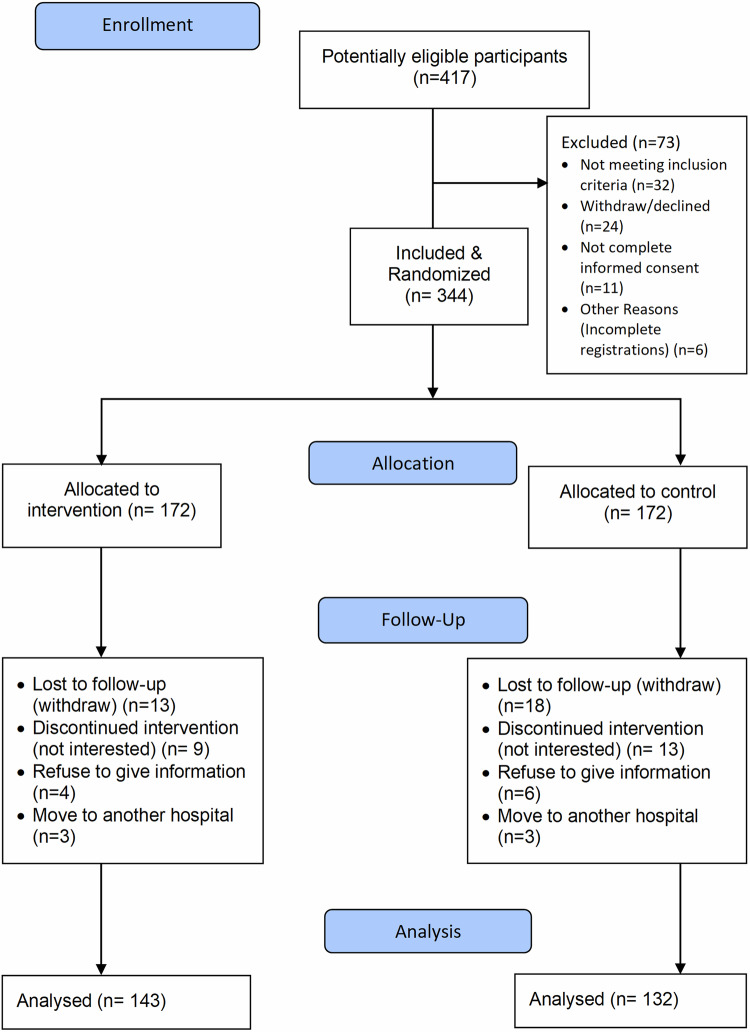
CONSORT flowchart of Participant’s Enrolment. Flow diagram showing screening, randomisation (Intervention N=143, Control N=132), losses and reasons at each follow-up (F1, F2) and numbers included in the primary analyses (intention-to-treat).

Table 2 illustrates that intervention (n = 143) and control (n = 132) groups were similar in age, gender, weight and baseline MMAS scores (*P* > *0.05*). Significant differences were noted in race (*P* = *0.03*), education (*P* = *0.04*), family size (*P* = *0.01*), and smartphone price (RM 1218.18 vs. RM 1118.26, *P* = *0.02*). At follow-up, the intervention group showed higher adherence (MMAS-F2: 6.50 vs. 5.50, *p* < 0.001) and better blood pressure outcomes (SBP:136.13 vs. 147.28 mmHg; DBP: 71.60 vs. 76.61 mmHg; both *P* < *0.001*). Satisfaction with the application was neutral (MASS: 3.65 ± 0.57) (Fig. 4).

**Table 2:** Participant Characteristics Categorized by Treatment Group.

Characteristic	Intervention N = 143 (52%)	Control N = 132 (48%)	*P* value
Age, mean (SD), y	64.01 (13.82)	65.02 (13.93)	0.57
Gender			0.80
Male, No. (%)	65 (45.5)	58 (43.9)	
Female, No. (%)	78 (54.5)	74 (56.1)	
Weight, mean (SD), kg	71.05 (18.23)	69.26 (15.22)	0.53
Race/ethnicity, No. (%)			0.03
Malay	63 (44.1)	40 (30.3)	
Chinese	50 (35.0)	65 (49.2)	
Indian	30 (21.0)	27 (20.5)	
Income, No. (%)			0.11
No Income	0 (0.0)	1 (0.8)	
B40 (RM 1 to RM 4849)	29 (20.3)	29 (22.3)	
M40 (RM 4850 to RM10959)	95 (66.4)	93 (71.5)	
T20 (RM 10960 & above)	19 (13.3)	7 (5.4)	
Education Level, No. (%)			0.04
No Formal Education	0 (0.0)	1 (0.8)	
Primary Education	9 (6.3)	19 (14.4)	
Secondary Education	73 (51.0)	71 (53.8)	
Tertiary Education	61 (42.7)	41 (31.1)	
Number of Family Members, No. (%)			0.01
1-3	48 (33.8)	66 (51.2)	
4-6	88 (62.0)	56 (43.4)	
7-9	6 (4.2)	7 (5.4)	
Smart Phone Price, mean (SD), RM	1218.18 (377.98)	1118.26 (351.29)	0.02
Adherence (MMAS), median (IQR)			
Baseline MMAS Score	5.25 (0.75)	5.25 (1.00)	0.13
F1 MMAS Score	5.75 (1.00)	5.50 (1.00)	<0.001
F2 MMAS Score	6.50 (1.00)	5.50 (1.19)	<0.001
Clinical Outcome (Blood Pressure SBP/DBP), mean (SD)			
Baseline Hypertension	146.23 (19.21)/ 77.08 (11.51)	142.01 (18.64) /75.89 (9.22)	0.09/0.39
Hypertension F1	138.69 (16.80) /74.75 (10.38)	142.79 (17.45) /74.01 (11.32)	0.07/0.60
Hypertension F2	136.13 (16.15) /71.60 (11.16)	147.28 (17.99) /76.61 (10.22)	<0.001/<0.001
Satisfaction, MASS score, median (IQR)			<0.001
User Interface	3.67 (1.33)		
Perceived Utility	3.50 (1.15)		
System Quality	4.00 (1.00)		
Service Quality	3.50 (1.00)		
Feature Satisfaction	3.38 (0.97)		
General Satisfaction	4.00 (1.34)		
MASS Score	3.65 (0.57)		

*SD* standard deviation, *IQR* inter-quartile range, *RM* malaysian ringgit, *SBP* systolic blood pressure, *DBP* diastolic blood pressure, *MMAS* morisky medication adherence scale, *MASS* mobile adherence satisfaction scale.

### SEM analysis

Structural Equation Modeling (SEM) was employed to investigate the interrelationships among the intervention, medication adherence, demographic variables, and clinical outcomes, with adherence functioning as a central mediating construct (Fig. 3). The model also incorporated demographic variables as potential moderators to evaluate their influence on outcome variables. Model fit was assessed using established goodness-of-fit indices, while path coefficients were analyzed to determine the statistical significance of the hypothesized associations (Table 1). Additionally, Necessary Condition Analysis (NCA) was utilized to identify critical antecedent factors required for achieving high levels of adherence and favorable clinical outcomes.

### Measurement model assessment

The construct’s reliability and validity were evaluated using SmartPLS 4 following Hair (2006) guidelines. Reliability was assessed via Cronbach’s alpha (internal consistency) and Composite Reliability (CR) [28].

As reported in Table 3, all constructs met the recommended thresholds for reliability and convergent validity. Cronbach’s α and composite reliability (CR) values exceeded 0.80 for all multi-item constructs, indicating strong internal consistency [28]. Average variance extracted (AVE) ranged from 0.62 to 0.78, confirming adequate convergent validity. All indicator loadings (λ) were above 0.73 and variance inflation factors (VIF) were below 3.03, suggesting no multicollinearity concerns [29].

**Table 3:** Reliability and convergent validity assessment.

Dimension	Items	λ	α	CR	AVE	VIF
Adherence (MMAS)		1.00				1.00
Intervention		1.00				1.00
Clinical Outcomes	SBP	0.90	0.53	0.80	0.67	1.15
	DBP	0.73				1.15
Satisfaction	UI	0.84	0.83	0.90	0.75	1.96
	PU	0.78	0.85	0.89	0.62	1.89
	SysQual	0.83	0.86	0.90	0.69	2.17
	ServQual	0.86	0.82	0.89	0.74	1.86
	FS	0.83	0.93	0.95	0.70	3.03
	GS	0.88	0.87	0.92	0.78	2.30

*SD* standard deviation, *CR* composite reliability, *AVE* average variance extracted, *VIF* variance inflation factor.

Discriminant validity was supported by both the Fornell–Larcker criterion and Heterotrait–Monotrait (HTMT) analysis (Table 4). The square roots of AVE exceeded their corresponding inter-construct correlations (above the diagonal), consistent with the Fornell–Larcker criterion (e.g., FS = 0.84, GS = 0.89, UI = 0.87; all greater than their correlations with other constructs, such as FS–PU = 0.37 and GS–UI = 0.31) [30]. HTMT values were all below the conservative threshold of 0.85, indicating sufficient discriminant validity (e.g., FS–PU = 0.21, GS–SysQual=0.34, Adherence–UI = 0.30) [31].

**Table 4:** Fornell–Larcker criterion and Heterotrait–Monotrait ratio of correlations.

	FS	GS	Hypertension Outcomes	Intervention	Adherence (MMAS)	PU	ServQual	SysQual	UI
FS	**0.84**	0.33	0.03		0.37	0.37	0.31	0.16	0.38
GS	0.36	**0.89**	0.02		0.21	0.20	0.24	0.33	0.31
Clinical Outcomes	0.06	0.08	**0.82**	0.29	0.28	0.03	0.06	0.05	0.07
Intervention			0.40	**1.00**					
Adherence (MMAS)	0.38	0.21	0.37	0.39	**1.00**	0.24	0.24	0.21	0.30
PU	0.21	0.24	0.08		0.24	**0.79**	0.25	0.36	0.35
ServQual	0.34	0.28	0.13		0.26	0.29	**0.86**	0.22	0.29
SysQual	0.16	0.34	0.11		0.20	0.41	0.27	**0.83**	
UI	0.41	0.36	0.10		0.33	0.40	0.34	0.41	**0.87**

Diagonal and italicized are the square roots of the AVE. Above the diagonal elements are the Fornell–Larcker ratio of correlations between the construct’s values. Below the diagonal elements are the Heterotrait–Monotrait ratio of correlation values.

Table 5 presented 34% of the variance of intervention in adherence (*R²*=0.34, *f²*=0.04) modest explanatory power [32, 33].

**Table 5:** Explanatory power using PLSpredict indicators.

Predictor	Exogenous Variable	*R*^*2*^	*f*^2^
Adherence (MMAS)	Intervention	0.34	0.04
Clinical outcomes		0.12	0.05

Table 6 shows SEM results where intervention had significant positive effects on medication adherence at both 3 months (β = 0.58, *P* < *0.001*) and 6 months (β = 0.79, *P* < *0.001*), with corresponding necessity effects in NCA (d = 0.18, *P* = *0.03*; d = 0.13, *P* < *0.001*), confirming that the intervention was not only sufficient but also necessary to achieve improved adherence [34]. Six-month adherence significantly predicted better hypertension outcomes (β = –0.20, *P* = *0.002*; d = 0.02, *P* < *0.001*), while the intervention also had a direct effect on clinical outcomes at 6 months (β = –0.44, *P* < *0.001*; d = 0.01, *P* < *0.001*), indicating that although the effect size was small in NCA, the condition was critical for improvement.

**Table 6:** Results of structural model path coefficients.

Hypotheses	Relationship	SEM					Necessity Conditions Analysis (LV SEM)	
		β	SD	T Statist ics	*P* Values	BI [2.5%;97.5%]	Necessity Effect Size (d)	Permutation *P* Values
H1+	Intervention → MMAS F1	0.58	0.11	5.30	<0.001	0.35; 0.78	0.18	0.03
H2+	MMAS F1 → Hypertension F1	-0.12	0.07	1.62	0.11	-0.23; 0.08	0.10	0.69
H3+	Intervention → Hypertension F1	-0.12	0.20	0.61	0.54	-0.41; 0.35	0.04	0.27
H4+	Intervention → MMAS F2	0.79	0.14	5.68	<0.001	0.52; 1.05	0.13	<0.001
H5+	MMAS F2 → Hypertension F2	-0.20	0.06	3.14	0.002	-0.32; -0.07	0.02	<0.001
H6+	Intervention → Hypertension F2	-0.44	0.13	3.42	<0.001	-0.67; -0.16	0.01	<0.001
H7	Demographics × Intervention → MMAS F2							
H7a-	Age × Intervention → MMAS F2	-0.16	0.10	1.63	0.10	-0.35; 0.03	0.05	0.002
H7b	Gender × Intervention → MMAS F2	-0.46	0.19	2.38	0.02	-0.83; -0.08	0.01	<0.001
H7c-	Weight × Intervention → MMAS F2	0.12	0.10	1.12	0.26	-0.09; 0.31	0.05	<0.001
H7d	Race × Intervention → MMAS F2	-0.01	0.10	0.05	0.96	-0.20; 0.18	0.04	0.001
H7e+	Income × Intervention → MMAS F2	-0.06	0.12	0.56	0.58	-0.27; 0.19	0.04	0.01
H7f+	Education × Intervention → MMAS F2	0.08	0.10	0.81	0.42	-0.12; 0.27	0.09	<0.001
H7g+	Number of Family × Intervention → MMAS F2	0.05	0.10	0.53	0.59	-0.15; 0.25	0.04	0.004
H7h+	Smartphone Price × Intervention → MMAS F2	-0.17	0.13	1.29	0.20	-0.45; 0.07	0.04	<0.001
H8+	Satisfaction → MMAS F2							
H8a+	UI → MMAS F2	0.10	0.06	1.58	0.12	-0.02; 0.22	0.29	0.02
H8b+	PU → MMAS F2	0.03	0.05	0.52	0.61	-0.08; 0.12	0.16	0.18
H8c+	SysQual → MMAS F2	0.04	0.06	0.72	0.47	-0.07; 0.14	0.25	0.07
H8d+	ServQual → MMAS F2	0.06	0.05	1.04	0.30	-0.05; 0.16	0.12	0.47
H8e+	FS → MMAS F2	0.20	0.06	3.31	<0.001	0.08; 0.31	0.29	0.005
H8f+	GS → MMAS F2	-0.02	0.05	0.40	0.69	-0.12; 0.07	0.07	0.37
H9+	MMAS F1 → MMAS F2	0.39	0.06	6.67	<0.001	0.28; 0.51	0.27	<0.001
H10+	Intervention → MMAS F1 → MMAS F2	0.22	0.05	4.33	<0.001	0.14; 0.35	-	-
H11+	MMAS F1 → MMAS F2 → Hypertension F2	-0.08	0.03	2.88	0.004	-0.14; -0.03	-	-
H12+	Intervention → MMAS F2 → Hypertension F2	-0.16	0.06	2.75	0.006	-0.28; -0.06	-	-
H13+	Intervention → MMAS F1 → MMAS F2 → Hypertension F2	-0.05	0.02	2.45	0.01	-0.09; -0.02	-	-

*SD* standard deviation, *BI* bias-corrected confidence interval, *LV SEM* latent variable structural equation modeling.

Adherence at 3 months was a strong predictor of 6-month adherence (β = 0.39, *P* < *0.001*; d = 0.27, *P* < *0.001*), confirming the temporal mediation pathway. Among satisfaction domains, only feature satisfaction (FS) was significantly associated with 6-month adherence in SEM (β = 0.20, *P* < *0.001*) and emerged as a necessary condition in NCA (d = 0.29, *P* = *0.005*). Other satisfaction variables (e.g., PU, GS, ServQual) did not show significant effects in SEM nor strong necessity in NCA.

Among demographic moderators, only gender significantly influenced the intervention–adherence link (β = –0.46, *P* = *0.02*), while NCA confirmed its necessity (*d* = 0.01, *P* < *0.001*). Age and weight showed moderate necessity effects (d = 0.05) despite being non-significant in SEM. Several mediation pathways were supported in SEM, including the indirect effects of the intervention on hypertension outcomes via adherence at both time points (e.g., H10–H13, all *P* < *0.01*), aligning with the proposed sequential mechanisms of change.

### Combined importance-performance map analysis (cIPMA)

cIPMA integrates strategic importance (IPMA) and necessity-based (NCA) perspectives into a unifying analysis framework to prioritize actionable levers based on identified determinants of adherence (MMAS F2) and clinical outcomes (Hypertension F2) at the end of study [35]. Importance reflects each construct’s total effect on the outcome, while performance indicates its observed level (0–100 scale) [36]. Constructs with high importance but low performance signal critical improvement areas. Additionally, CE-FDH values from NCA highlight conditions necessary for achieving high adherence or clinical outcomes [37]. This dual approach enables prioritization of constructs that are both influential and indispensable, thereby guiding targeted enhancements in digital adherence interventions (Table 7).

**Table 7:** cIPMA results of adherence and clinical outcomes.

	Adherence (MMAS F2)					Clinical Outcomes (Hypertension F2)				
Antecedent Construct	IPMA		NCA (LV IPMA)			IPMA		NCA (LV IPMA)		
	Importance	Performance	Necessary condition			Importance	Performance	Necessary condition		
	Standardized Total Effects		CE-FDH (100%)	Original Effect Size (d)	Permutation *P* Values	Standardized Total Effects		CE-FDH (100%)	Original Effect Size (d)	Permutation *P* Values
Intervention Type	0.77	52.00	100	0.001	<0.001	0.60	52.00	100	0.001	<0.001
MMAS F1	0.38	58.99	70.58	0.28	<0.001	0.20	58.99	82.35	0.12	0.24
MMAS F2	-	-	-	-	-	0.37	48.12	73.33	0.02	0.73
Hypertension F1	0.10	54.62	34.08	0.10	0.71	0.49	56.77	48.58	0.26	<0.001
Hypertension F2	-	-	-	-	-	-	-	-	-	-
UI	0.09	66.18	81.52	0.29	0.01	0.02	66.47	66.46	0.16	0.24
PU	0.04	62.58	52.15	0.16	0.18	0.03	59.36	59.35	0.17	0.01
SysQual	0.03	70.60	67.58	0.25	0.07	0.10	70.72	70.72	0.17	0.20
ServQual	0.06	50.08	52.92	0.12	0.50	0.03	50.09	50.09	0.15	0.09
FS	0.20	56.58	57.48	0.28	0.005	0.13	56.26	56.26	0.16	0.08
GS	0.02	50.35	45.99	0.06	0.35	0.05	16.67	16.674	0.10	0.23

*LV IPMA* latent variable importance-performance map analysis, *NCA* necessary condition analysis, *CE-FDH* ceiling-envelopment using the free disposal hull algorithm.

### cIPMA results of MMAS F2

**Fig. 5:**
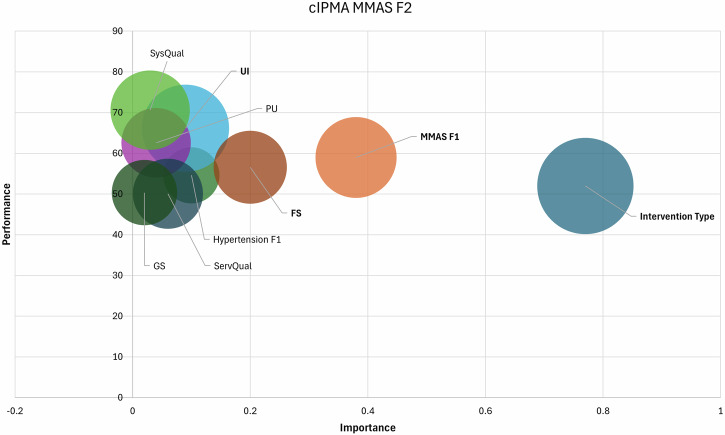
Combined Importance–Performance Map of the MMAS F2. cIPMA scatterplot with importance (x-axis) and performance (y-axis) for antecedents of MMAS F2; markers sized by CE-FDH necessity and labelled for Intervention, Hypertension F1 (prior BP control), MMAS F1, UI, FS, SysQual, ServQual, PU, GS. The shaded quadrant (high importance, low performance) marks priority targets for optimization.

The intervention exhibited the highest importance (β = 0.77) and moderate performance (52.00), while being a perfect necessary condition (CE-FDH = 100%, *d* = 0.001, *P* < *0.001*) for achieving high levels of MMAS F2 (Table 7). Early adherence (MMAS F1) also emerged as a critical enabler, showing moderate importance (0.38), high performance (58.99) and strong necessity (CE-FDH = 70.58, *d* = 0.28, *P* < *0.001*). This indicated that achieving optimal MMAS F2 depends significantly on prior adherence behavior. Among satisfaction domains, User Interface (UI) and Feature Satisfaction (FS) had moderate importance (0.09 and 0.20) and high performance (66.18 and 56.58), but importantly, they were statistically significant necessary conditions (UI: CE-FDH = 81.52, *d* = 0.29, *P* = *0.01*; FS: CE-FDH = 57.48, *d* = 0.28, *P* = *0.005*). These findings suggest that without sufficient usability and functionality, high adherence is unlikely, regardless of other factors (Fig. 5).

### cIPMA results of hypertension F2

**Fig. 6:**
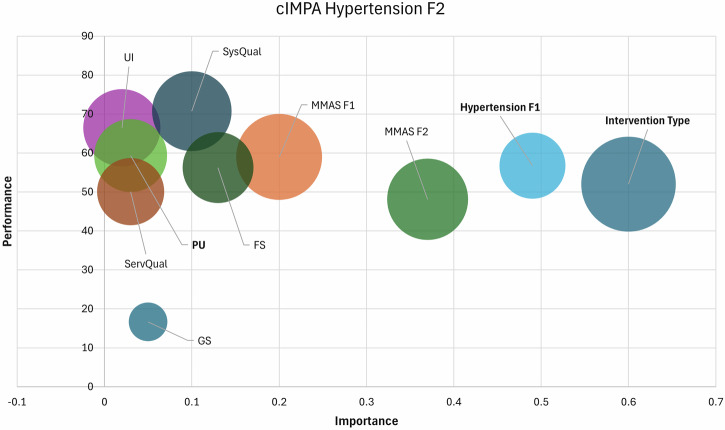
Combined Importance–Performance Map of the Hypertension F2. cIPMA scatterplot for predictors of Hypertension F2 showing Intervention, Hypertension F1 (prior BP control), MMAS F1/F2 and satisfaction domains; CE-FDH necessity statistics are annotated for key predictors. The shaded quadrant (high importance, low performance) marks priority targets for optimization.

The intervention type exhibited the highest importance (β = 0.60) with moderate performance (52.00) and a CE-FDH value of 100, indicating it was both a high-impact predictor and a perfect necessary condition (*d* = 0.001, *P* < *0.001*) for achieving improved blood pressure outcomes at six months (Table 7). Prior blood pressure control (Hypertension F1) was another strong contributor (importance=0.49; performance=56.77) and was also a significant necessary condition (CE-FDH = 48.58, *d* = 0.26, *P* < *0.001*), highlighting the importance of baseline clinical control in determining future BP levels. Among satisfaction constructs, only PU reached statistical significance as a marginal necessary condition (CE-FDH = 59.35, *d* = 0.17, *P* = *0.01*) (Fig. 6).

## Discussion

This study provides strong evidence that the Mobile Adherence Intervention significantly enhances medication adherence and blood pressure control among hypertensive patients in Malaysia’s middle-income setting. More importantly, by integrating SEM, NCA and cIPMA, we move beyond conventional efficacy reporting to elucidate the causal mechanisms, necessary preconditions and optimization priorities underlying digital adherence interventions.

Our findings validate the core theoretical proposition that adherence operates as a temporally sequenced process, where early behavioral gains (3-month adherence) serve as the critical foundation for sustained habit formation and downstream clinical improvement at 6 months. The intervention demonstrated strong direct effects on both short-term and long-term adherence. Crucially, SEM revealed that early adherence (MMAS F1) strongly predicted later adherence, while serial mediation analyses confirmed that the intervention’s downstream impact on 6-month clinical outcomes operated primarily through this temporal pathway. This empirically supports our integration of the EMERGE framework’s initiation-implementation paradigm and Self-Regulation Theory, highlighting that successful mHealth interventions must first secure early engagement to catalyze long-term behavior change [18].

The sequential mediation pattern supports the Temporal Mediation Paradigm and aligns with contemporary habit-formation literature showing that initial behaviour change must be consolidated to sustain long-term adherence [38]. Consistent with the COM-B model, CareAide strengthened capability (digital pillbox, dose history), opportunity (contextual reminders) and motivation (progress feedback), thereby overcoming well-documented barriers such as forgetfulness and regimen complexity [39]. Two recent meta-analyses and several single-centre RCTs in Asia and Latin America report pooled improvements of 0.7–1.2 MMAS points and 4–10 mmHg SBP with smartphone interventions, mirroring our effect sizes [40, 41].

Consistently, a 2025 meta-analysis of 76 studies confirmed that smartphone application-based interventions significantly reduced systolic (–2.8 mmHg) and diastolic (–1.2 mmHg) blood pressure at six months [42]. However, most prior studies relied solely on pre–post comparisons or binary adherence thresholds; the present trial adds nuance by modelling temporal mediation and sufficiency/necessity relationships.

The application of NCA yielded deeper insights, revealing that the intervention itself was not merely sufficient but necessary for achieving high adherence. Similarly, early adherence emerged as a non-negotiable precondition for long-term adherence success. This necessity perspective, largely absent in prior mHealth literature implies that in real-world contexts, high adherence is unlikely to occur without such digital scaffolding, particularly among patients with established non-adherence. Interestingly, while user satisfaction overall was neutral, only feature satisfaction encompassing functionality like reminder reliability and tracking accuracy significantly predicted sustained adherence and was deemed necessary by NCA [43]. This was congruent with the Unified Theory of Acceptance and Use of Technology, wherein performance expectancy and hedonic experience drive sustained engagement, suggesting that in chronic disease management, practical functional utility outweighs interface aesthetics or perceived ease of use [44]. System and service-quality domains were non-significant, echoing recent reviews that report diminishing marginal returns from generic reliability attributes once baseline technical stability is achieved [45]. The limited influence of other satisfaction domains (e.g., system quality, general satisfaction) implies that designers should prioritize core functionalities that directly reduce adherence barriers (forgetfulness, regimen complexity) over peripheral enhancements [46].

Sociodemographic moderation analysis revealed nuanced boundary conditions. Gender significantly moderated intervention effectiveness, with males deriving greater benefits, a finding corroborated by NCA. While SEM showed non-significant effects for age, weight and education, NCA indicated their marginal necessity, suggesting complex interactions between digital literacy, comorbidities and engagement. Baseline disparities in education, larger family size and smartphone cost highlight potential access barriers for lower socioeconomic groups [47], showing the need for equitable implementation strategies, consonant with national surveys linking low digital literacy to reduced mHealth usability [48, 49].

The cIPMA synthesis provides actionable guidance for optimizing future interventions. The intervention’s combination of high strategic importance for sustained adherence and perfect necessity confirms its foundational role [50]. However, its moderate performance signals room for enhancement, particularly in accessibility for resource-constrained users. cIPMA suggests that investment in interface refinements and personalized feature bundles could yield the largest adherence gains, while NCA highlights the indispensability of early-phase adherence coaching supporting staged implementation strategies that intensify support during the first three months. Feature satisfaction and early adherence were identified as high-priority optimization targets: both demonstrated high necessity and moderate-to-high importance, yet their performance was suboptimal [24, 51]. This suggests that embedding intensified early-phase support (e.g., proactive clinician alerts during the first month, gamified feedback loops) and feature refinements (e.g., adaptive reminders, simplified data entry) could yield substantial improvements. The minimal influence of baseline blood pressure on adherence pathways, contrasted with its necessity for clinical outcomes (Hypertension F1), reinforces that digital tools complement but do not replace biomedical management.

### Limitations and future directions

Generalizability may be constrained by the single-center, urban sampling and smartphone ownership requirement, potentially excluding digitally marginalized populations. While the 6-month duration captured initiation and implementation phases, long-term maintenance ( > 1 year) remains unverified. Future research should test adapted interventions in rural settings, incorporate objective adherence metrics (e.g., electronic monitoring) and explore integration with Malaysia’s public health infrastructure.

## Conclusion

This study demonstrates that the CareAide application effectively addresses hypertension medication non-adherence in Malaysia by utilizing temporal behavioral mechanisms and functional reinforcement. By identifying the intervention and early adherence as necessary conditions and featuring satisfaction as a both sufficient and necessary lever for optimization, we provide a replicable framework for developing theory-driven, equitable digital health solutions. Prioritizing user-centered functionality over aesthetic appeal, ensuring proactive early-phase engagement and addressing socioeconomic access barriers will be critical for scaling impact. By uniting behavioral theory with advanced sufficiency-necessity analytics, this study furnishes a roadmap for optimizing and scaling digital adherence solutions across similar middle-income contexts beyond traditional efficacy metrics.

## Summary

### What is known about topic

- Medication non-adherence is a contributor to suboptimal blood pressure control and poor cardiovascular outcomes in hypertensive populations, especially in middle-income countries.
- mHealth interventions can improve adherence but are often evaluated without theory-based frameworks.
- The mechanisms linking application use to clinical outcomes are poorly understood.
- User satisfaction is rarely modeled as a determinant of adherence behavior.

### What this study adds

- Early adherence mediates the effect of mHealth interventions on long-term clinical outcomes.
- Mobile adherence application usage, early adherence and feature satisfaction with application are necessary conditions for achieving sustained medication adherence and improved blood pressure outcomes.
- A novel triangulated approach combining SEM, NCA and cIPMA to reveal causal pathways and optimization priorities for designing effective mobile adherence application.

## Data Availability

The datasets generated during and/or analyzed during the current study are available from the corresponding author upon reasonable request.
